# P02.167. Long term evaluation of homeopathy on post treatment impairment of pulmonary tuberculosis

**DOI:** 10.1186/1472-6882-12-S1-P223

**Published:** 2012-06-12

**Authors:** S Sharma, N Sharma

**Affiliations:** 1 NMP Medical Research Institute, Jaipur, India; 2 Brett Research (UK), London, United Kingdom

## Purpose

Previous studies show that treated and cured pulmonary tuberculosis patients do suffer from pulmonary impairment, lower health related quality of life, disability and long term morbidity, thus responsible for a majority of the disease burden. Despite this, no effective management is available for most of the patients. Therefore, the present study was undertaken to evaluate the impact of homeopathy on pulmonary, functional and quality of life status of patients with pulmonary tuberculosis who have completed treatment.

## Methods

Patients who were cured and had completed anti-tuberculosis treatment within a period of 5 years were enrolled in a randomized double-blind placebo-controlled trial. Individualised homeopathy treatment was given to 61 patients and identical placebo to 57 patients. Symptomatic changes, pulmonary function tests, and health related quality of life were assessed prior to treatment, after 6 months of intervention, and followed up for a year after completing the intervention.

## Results

Significant improvement was observed with the homeopathy treatment in FEV1 (p<0.001), forced vital capacity (p<0.001), and FEV1/FVC ratio (p=0.002). Symptom scores for cough and breathlessness were significantly lower with homeopathy than with placebo (p<0.001). At the end of treatment, patients on homeopathy had increased body weight (p<0.0001), and better quality of life (p<0.05) compared with placebo (p=0.003). Benefits were maintained in the homeopathy group after a year whereas symptoms (p<0.01) and impact score (p<0.001) deteriorated in placebo. Physicians visits were reduced in the homeopathy group by 58.0% (p =0.002) compared to placebo (p<0.0001).

## Conclusion

Homeopathy is effective in improving lung capacity and health status. Benefits remain evident after a year. This suggests that homeopathy could make an important contribution to post treatment tuberculosis pulmonary impairment.

